# The dimensionality of fatigue in Parkinson’s disease

**DOI:** 10.1186/s12967-018-1554-z

**Published:** 2018-07-11

**Authors:** Raymond Chong, Lauren Albor, Chandramohan Wakade, John Morgan

**Affiliations:** 1 Department of Interdisciplinary Health Sciences, Augusta University, Augusta, Georgia USA; 2 Cincinnati Children’s Hospital Medical Center, Cincinnati, Ohio USA; 3 Department of Neurology, Augusta University, Augusta, Georgia USA; 4 Augusta VMAC, Augusta, Georgia USA

**Keywords:** Canonical correlation, Discrimination function analysis, Factor analysis, Visual analog

## Abstract

**Background:**

Fatigue is a common problem among individuals with Parkinson’s disease (PD). It may occur before the overt symptoms of bradykinesia, rigidity and tremor. Little is understood about how to measure fatigue in PD. Here we determined the dimensionality of the constructs of fatigue.

**Methods:**

Four recommended scales, the Fatigue Severity Scale, Functional Assessment of Chronic Illness Therapy-Fatigue, Parkinson Fatigue Scale and Visual Analog Fatigue Scale (VAFS) were tested against quality of life measures including cognition, depression, sleep, life orientation, physical activity and PD symptoms in 22 PD subjects and 15 caregivers.

**Results:**

Fatigue was associated with many quality of life variables, with the PDQ-39 summary index showing the strongest association. PD subjects agreed more strongly than caregivers that they experienced higher levels of fatigue. 27% of PD subjects rated fatigue as one of their top three most bothersome symptoms. The constructs of fatigue was captured within one dimension which explained 67% of the total variance, of which the VAFS showed the highest internal consistency. The highest likelihood ratio gave a cut-off score of < 5.5 on the VAFS. The change in scores required to produce a perceptible difference or is grossly observable ranged between 1.4 and 2.2 points respectively.

**Conclusion:**

The potential utility of a single measure such as the VAFS in PD that is reliably correlated with quality of life is consistent with the pursuit to develop clinical tests and measurements that are accessible, easy to use and universally interpretable across health science disciplines.

## Background

Between 30 and 70% of individuals with Parkinson’s disease (PD) experience undue fatige everyday [1–4]. It is a symptom which may appear several years before overt symptoms prompt the disease diagnosis. Although it significantly impacts quality of life, understanding its origin or mechanisms has remained elusive. Early evidence also suggests that fatigue in PD is not due to dopamine deficiency. Instead, the serotonin system and possibly autonomic disorder may play a role. Pharmaceutical interventions such as Rasagiline [5, 6], doxepin and modafanil [7] provide some relief but do not prevent the symptom from progressing.

No evidence-based guidelines are available to treat fatigue in PD. So little is known about the symptom that it is not even clear how to classify it, whether it should be a central, physical, cognitive, peripheral or mixed/complex phenomenon. Researchers and clinicians have attempted to document the perception of fatigue among PD patients through surveys. Many scales have been used, of which nine of the most commonly ones were reviewed by the Movement Disorders Society (MDS) task force [3]. Since not all the scales were developed for PD, recommendations were based on available reports to estimate their validity for use in this population. The scales were judged on their ease of use, namely the number of items and time needed to administer the questions. All the scales were rated as Listed (i.e., acceptable), Suggested or Recommended. None received a Not-Recommended rating. Neither was any scale given a Highly-Recommended ranking. Thus, despite best attempts to come up with a method of quantifying the perception of fatigue in PD, it remains unclear what the best way is to implement it.

Here in this pilot study, we sought to examine the dimensionalities of the fatigue scales reviewed by the MDS task force. We wanted to determine whether consolidation of these commonly used scales may be achieved, or abandon them and instead develop an entirely new scale. Importantly, we wanted to also determine how the assessment of fatigue relates to willingness to engage in activities of daily living. It is intuitive to reason that higher levels of fatigue perception negatively impacts quality of life. We looked at three scales rated as Recommended for screening fatigue in PD by the task force: the Fatigue Severity Scale (FSS), the Functional Assessment of Chronic Illness Therapy-Fatigue Scale (FACIT-F) and the Parkinson Fatigue Scale (PFS). In addition, we looked at the Fatigue Visual Analog Scale (VAFS). This one-item interval scale was rated as acceptable. It did not receive a higher rating due to lack of research regarding its sensitivity to change. Nevertheless, we included it because it is the simplest crude test for assessing fatigue perception. It has been used to validate a number of other PD fatigue scales. Our hope is to enable better documentation of fatigue in PD through the use of as few items as possible that are highly correlated with quality of life measures. Our goal is also consistent with the quest to develop measurements that are easily accessible, easy to use and universally interpretable across the health science disciplines [8].

## Methods

### Participants

A convenience sample of 37 subjects participated in the study which was approved by the Institution’s human-subjects review board. 22 subjects were individuals diagnosed with idiopathic Parkinson’s disease PD (69 ± 11 years old, 15 men, 7 women, H&Y median = 2). These were patients attending the Movement Disorders Clinic. The remaining 15 subjects served as age-matched controls, the majority of whom were caregivers of the PD subjects (63 ± 9 years, 2 men, 13 women).

### Procedures

After informed consent was obtained, subjects in both groups completed the four fatigue scales: the Fatigue Severity Scale (FSS), the Functional Assessment of Chronic Illness Therapy-Fatigue Scale (FACIT-F), the Parkinson Fatigue Scale (PFS) and the Fatigue Visual Analog Scale (VAFS). PD subjects were then asked to list their top three most bothersome PD symptoms. They completed 13 quality of life-related measures, as follows:

- Cognition: Montreal Cognitive Assessment (MoCA)
- Anxiety/depression: Hospital Anxiety and Depression Scale (HADS)
- PD symptoms: Unified Parkinson’s disease Rating Scale (UPDRS)
- Three disease attributes: duration, age at diagnosis, body mass index
- Non-motor signs/symptoms: Non-Motor Symptom Questionnaire and Rating Scale (NMSS and NMSQ)
- Quality of life: Parkinson’s Disease Quality of Life Questionnaire-39 Summary Index (PDQ-39SI)
- Physical activity level: EPIC Physical Activity Questionnaire (ePAQ)
- Sleep: Epworth Sleepiness Scale (ESS) and PD Sleep Scale (PDSS)
- Optimism: life orientation test (LOT-R)

Caregivers (control subjects) were asked to rank the top three most bothersome symptoms that they think their loved ones experience. They also filled in the Caregiver Strain Index form.

### Analyses

We first tested the appropriateness of the four fatigue scales by checking their associations between the fatigue scores and the 13 quality of life measures with a canonical correlation analysis. We then ran the discriminant function test to determine how well the scales can distinguish between excessive fatigue and normal fatigue. Following these two analyses, we combined the scales and carried out a factor analysis to determine the number of dimensions from these items, the total explained variance and the internal consistency of their correlations. We then ran a receiver operating curve analysis on the variable which had the highest internal consistency reliability to determine the cut-off score. The area under the curve, along with 95% confidence intervals was calculated. The optimal cut-off score was determined by choosing the value that produced the highest likelihood ratio of a positive prediction of fatigue. This choice of cut-off gave the best balance between sensitivity and specificity. Sensitivity and specificity, including 95% confidence intervals were calculated based on these cut-offs. Finally, we estimated the minimum change in fatigue score that is needed to capture a reliable test–retest analysis.

## Results

### Association between fatigue and quality of life in Parkinson’s disease

To determine the relationship between the perception of fatigue and quality of life, a canonical correlation analysis was conducted using the fatigue scales as the criterion variables and quality of life measures as the predictor variables. The range of correlation within the fatigue scales was r = − 0.86 to 0.85 and r = − 0.74 to 0.82 among the quality of life variables. The highest correlated predictor variables was between the PDQ-39SI and NMSQ measures. Omission of either variable did not change the results; these two variables were therefore retained. The multivariate analyses produced four discriminant functions, of which the first was significant, F(13,8) = 13.24, p = 0.0005 based on Roy’s greatest-root test. The correlation between the two sets of variables was Rc = 0.98, indicating a high degree of relationship between the predictor and criterion variables. The squared canonical correlation which represented the proportion of the variance in the canonical variate of the fatigue scales that can be explained by the canonical variate of the quality of life variables was 96%.

The canonical loadings were then examined to determine which variables in each multivariate set contributed most substantively to the overall relationship between the two sets of variables. The loadings for the variables can be seen in Table 1. Variables with a loading of > 0.3 contributed significantly to the multivariate relationship. For quality of life, all variables except body mass index were significant, with the PDQ-39SI contributing the most. All the fatigue scales contributed importantly to the canonical correlation. The redundancy index revealed that 30% of the variance in the fatigue scales was explained by the quality of life measures (with > 10% considered as significant and meaningful). The combined results indicated that a significant relationship exists between perception of fatigue and quality of life.

**Table 1:** Canonical loadings for the fatigue (criterion) and quality of life (predictor) in the Parkinson group

Variables	Mean (SD)	Range	Loadings
Predictor variables (quality of life)			
1. PD Quality of Life Questionnaire Summary Index, PDQ-39SI	27 (20)	4–65	0.82
2. Non-Motor Symptoms Questionnaire, NMS-Q	12 (6)	1–21	0.73
3. Hospital Anxiety and Depression Scale, HADS	12 (7)	3–24	0.71
4. Non-Motor Symptoms Scale, NMSS	66 (44)	5–190	0.67
5. Unified Parkinson’s Disease Rating Scale Total, UPDRS	43 (14)	20–73	0.66
6. Duration of disease (years)	8 (5)	1–22	0.46
7. Epworth Sleepiness Scale, ESS	8 (4)	1–15	0.40
8. Age at diagnosis (years)	63 (9)	45–79	0.38
9. Body mass index, BMI	27 (7)	17–54	− 0.18
10. Life orientation test, LOT-R	17 (5)	5–24	− 0.40
11. Montreal Cognitive Assessment, MoCA	26 (4)	15–30	− 0.52
12. PD sleep Scale	100 (26)	42–144	− 0.53
13. EPIC Physical Activity Questionnaire, ePAQ	72 (68)	8–318	− 0.43
Criterion variables (Fatigue)			
1. Fatigue Severity Scale, FSS	38 (15)	11–60	0.86
2. Functional Assessment of Chronic Illness Therapy Fatigue Scale, FACIT-F	32 (12)	14–50	− 0.83
3. Parkinson Fatigue Scale, PFS	50 (17)	17–77	0.80
4. Visual Analog Fatigue Scale, VAFS	6 (2)	2–10	− 0.69

All quality of life variables except body mass index reliably predicted perception of fatigue in Parkinson’s disease. The PDQ-39SI produced the strongest prediction

### Severity of fatigue in Parkinson’s disease

Unlike the previous canonical correlation analyses, the four fatigue scales are considered predictor variables in this evaluation as they are being used to determine how well they classify participants as belonging to the Control or PD group. The correlations among the four scales ranged from − 0.89 to 0.77. We analyzed the data with and without the FACIT-F due to its high correlation (− 0.89) with the PFS. The discriminant function was significant, Wilks’ lambda = 0.63, F(4,32) = 4.79, p = 0.0038 with FACIT-F and 0.0013 without. Specifically, the Parkinson group agreed more strongly than the Control group that they experienced higher levels of fatigue. 27% of PD subjects (6/22) ranked fatigue as their top three most bothersome symptoms (Table 2).

**Table 2:**
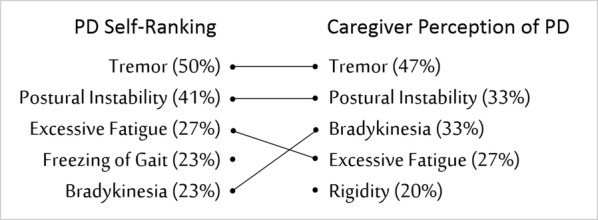
Ranking of bothersome PD symptoms

The standardized discriminant function coefficients (based on the pooled within-class standardized canonical coefficients) revealed that the PFS contributed the most to maximizing group differences, followed by the FSS and VAFS. The FACIT-F contributed negligibly to the difference in fatigue perception between the groups. Classification analysis indicated that, on the basis of the PFS, FSS and VAFS predictor variables, 93.3% of Control and 77.3% of PD participants could be correctly predicted to their respective group. Using base rates of 41.7 and 61.1% respectively, the accuracy of group prediction was improved over chance by 52 and 16% respectively. The standardized z test for the prediction rate was 4.8 with the FACIT-F and 4.7 without. Thus, the ability to predict subjects to the Control or PD group beyond chance was reliable. The means and standard error for all variables by group are summarized in Table 3.

**Table 3:** Mean and standard error of the fatigue scales and standardized discriminant function coefficients (SCFC)

Variable	Control mean (SE)	PD mean (SE)	SCFC
1. Parkinson Fatigue Scale, PFS	26.8 (3.6)	49.9 (3.6)	0.86
2. Fatigue Severity Scale, FSS	21.9 (3.6)	38.1 (3.1)	0.44
3. Visual Analog Fatigue Scale, VAFS	7.7 (0.7)	5.6 (0.5)	0.42
4. Functional Assessment of Chronic Illness Therapy Fatigue Scale, FACIT-F	43.9 (1.6)	31.5 (2.6)	− 0.09

The PD group agreed more strongly than the Control group that they experienced higher levels of fatigue. The standardized discriminant function coefficients (SCFC) revealed that the PFS scale contributed the most to classifying subjects as healthy controls or PD. The FACIT-F contributed the least to the classification

### Dimensionality of the fatigue scales

A factor analyses was carried out on the PFS, FSS and VAFS scales which were shown earlier in the discriminant function analysis to be reliable predictor variables The scree plot revealed that the Eigen value for the first factor was 7.5, 15 points higher than the second factor of 2.3. Given the high first Eigen value, we estimated the common dimensionality of the three fatigue scales by extracting a one-factor solution comprising all the items from the three scales. The single solution accounted for 67% of the total variance, suggesting that the items from the three scales were unidimensional. We next checked the scale reliability of the items by examining their internal consistency. The overall standardized alpha coefficient was 0.97. All items scored > 0.97, suggesting that they were all highly consistent. The most reliable item was the VAFS, with a coefficient of 0.98 (Table 4).

**Table 4:** Simple statistics and standardized Cronbach coefficient alpha of the fatigue scales

Item	Label	Mean (SD)	Range	Standardized Cronbach coefficient alpha
Fatigue Severity Scale (FSS)				
1	My motivation is lower when I am fatigued	4.3 (1.9)	1–7	0.969
2	Exercise brings on my fatigue	3.2 (1.7)	1–7	0.970
3	I am easily fatigued	3.8 (2.3)	1–7	0.967
4	Fatigue interferes with my physical functioning	3.4 (2.0)	1–7	0.968
5	Fatigue causes frequent problems for me	3.1 (2.2)	1–7	0.967
6	My fatigue prevents sustained physical functioning	3.3 (2.2)	1–7	0.967
7	Fatigue interferes with carrying out certain duties and responsibilities	3.4 (2.1)	1–7	0.967
8	Fatigue is among my most disabling symptoms	3.4 (2.4)	1–7	0.967
9	Fatigue interferes with my work, family, or social life	3.6 (2.3)	1–7	0.967
Parkinson Fatigue Scale (PFS)				
1	I have to rest during the day	3.0 (1.4)	1–5	0.968
2	My life is restricted by fatigue	2.5 (1.4)	1–5	0.967
3	I get tired more quickly than other people I know	2.9 (1.5)	1–5	0.967
4	Fatigue is one of my three worst symptoms	2.8 (1.6)	1–5	0.967
5	I feel completely exhausted	2.3 (1.4)	1–5	0.967
6	Fatigue makes me reluctant to socialise	2.2 (1.4)	1–5	0.968
7	Because of fatigue it takes me longer to get things done	3.0 (1.5)	1–5	0.967
8	I have a feeling of ‘heaviness’	2.2 (1.3)	1–5	0.968
9	If I wasn’t so tired I could do more things	2.8 (1.4)	1–5	0.967
10	Everything I do is an effort	2.3 (1.3)	1–5	0.967
11	I lack energy for much of the time	2.6 (1.3)	1–5	0.967
12	I feel totally drained	2.4 (1.3)	1–5	0.967
13	Fatigue makes it difficult for me to cope with everyday activities	2.4 (1.3)	1–5	0.967
14	feel tired even when I haven’t done anything	2.4 (1.3)	1–5	0.968
15	Because of fatigue I do less in my day than I would like	2.7 (1.4)	1–5	0.967
16	I get so tired I want to lie down wherever I am	2.1 (1.4)	1–5	0.967
Visual Analog Fatigue Scale (VAFS)				
–	Visual Analog Fatigue Scale	6.5 (2.7)	2–10	0.979

The one-factor solution comprising all the items of the three scales explained 67% of the total variance, suggesting that the items from the three scales were unidimensional. The overall standardized alpha coefficient was 0.97. The most reliable item was the VAFS, with a coefficient of 0.979

### Receiver operating curve analysis (ROC) of the Visual Analog Fatigue Scale

**Fig. 1:**
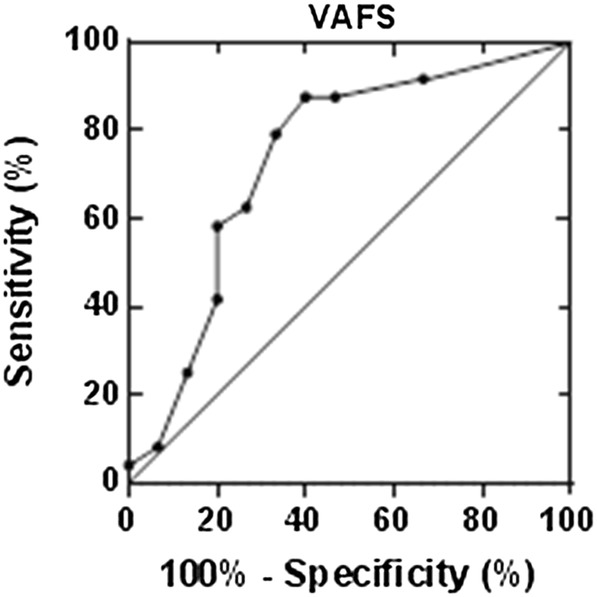
Receiver operating curve of the Visual Analog Fatigue Scale

The ROC analyses showed that subjects who rated their fatigue at < 5.5 were three times more likely to experience excessive fatigue associated with PD. A summary of the ROC analyses, including the area under the curve (AUC), sensitivity, specificity, and cut-off score based on the highest likelihood ratio is shown in Table 5, along with the corresponding 95% confidence intervals. A graph of the ROC can be seen in Fig. 1.

**Table 5:** Summary of the receiver operating curve analysis of the Visual Analog Fatigue Scale

Area under the curve	0.74 (0.09)
95% confidence interval	0.56–0.91
Cutoff score	< 5.5
Sensitivity %	58.3
95% confidence interval	36.6–77.9
Specificity	80.0
95% confidence interval	51.9–95.7
Likelihood ratio	2.9

### Sensitivity to change in the Visual Analog Fatigue Scale

Using the Cohen’s medium and large effect size values of 0.5 and 0.8 to estimate the effect of a change in perception of fatigue, the VAFS was estimated to differ between 1.4 and 2.2 points between a grossly observable change (medium effect) to a perceptible change (large effect) respectively.

### Caregiver strain

Caregivers’ perception of effort in taking care of their PD loved ones was 3.3 ± 3.5 on the Caregivers Strain Index. According to the index, the threshold for a high level of caregiver stress was ≥ 7 [9].

## Discussion

The new finding in this study relates to the identification of the VAFS as a potential reliable estimate of assessing the overall sensation of excessive fatigue experienced by individuals with PD. Our finding of the VAFS being a highly consistent and reliable measure of fatigue in PD is consistent with other studies which used a similar visual analog scale to evaluate other conditions of fatigue [10] and subjective report such as pain [11]. It is intuitive to realize that when a visual analog scale is used to document severity or a change in condition, the individuals being assessed are often basing their response on how they feel overall. Thus, although lengthy questionnaires that are specific and detailed may serve their purposes, it appears that a simple question asking subjects how they feel overall (such as fatigue or pain) succinctly captures the status of their well-being.

Our finding of high internal consistency among the 16 items from the Parkinson Fatigue Scale (PFS), Fatigue Severity Scale (FSS) and Visual Analogue Fatigue Scale (VAFS) is similar to a previous report on the Modified Fatigue Impact Scale (MFIS) [12]. The MFIS is a newer Parkinson fatigue scale and was therefore not included in the MDS task force’s reviews. The MFIS has 21 items which asks about cognitive, physical and non-physical components. Their analyses showed a two-factor structure, similar to the current analyses on the PFS, FSS and VAFS fatigue scales. Unfortunately, their Eigen values were not reported. Nevertheless, similar to the current study, the authors also found high internal consistency in all their 21 items of at least 0.95. Thus, despite the lack of consensus about the definition of fatigue in PD and the root cause of it, available evidence suggests that the constructs of fatigue in PD may be appreciated as unidimensional, at least in terms of how it impacts quality of life.

Despite our smaller sample, the results of the study appear to provide useful pilot data, out of which further understanding of fatigue in Parkinson’s disease (PD) may be advanced. The fatigue scales which we used in the study and recommended by the MDS task force were associated with many aspects of quality of life. Indeed, the PDQ-39 summary index was found to show the highest correlation with the fatigue scales, while possible confounders of fatigue such as daytime sleepiness and depression had lower correlations [13, 14]. Our findings are also consistent with published data on the prevalence of fatigue as well as the ranking of fatigue as the top three most bothersome PD symptoms.

Another potential limitation in the study is the use of caregivers as control subjects. Certainly, caregiver strain may produce undue fatigue and confound the study. In addition to the convenience in recruitment, there are several advantages of using caregivers as control subjects: (1) Examination of the data showed that only 3 subjects rated their fatigue at < 5 on the VAFS. The rest of the control subjects did not exceed the threshold for significant caregiver stress. We did not find it surprising that the control subjects reported low-level caregiver strain as our PD subjects were mostly in stage 2 of the H&Y and were therefore still ambulatory and living independently. Perhaps the use of the term caregiver may be a bit overstated in this case. (2) The second advantage was that by asking the spouses to estimate the severity of fatigue experienced by their PD spouse, we were able to determine that the spouses could accurately detect and perceive the severity of their PD spouse’ fatigue. (3) The third advantage of using caregivers as control subjects was that we were able to estimate how much more fatigue the PD subjects experienced compared to a family member who lived under similar housing/neighborhood conditions and lifestyles. Overall, the results showed that PD subjects experienced a lot more fatigue than their caregivers. The fatigue experienced by many of the PD subjects was excessive and unusual.

## Conclusion

The potential use of a single measure such as the VAFS in PD that is reliably correlated with quality of life measures is consistent with the pursuit to develop clinical tests and measurements that are accessible, easy to use and universally interpretable across the health science disciplines [8]. Although it may appear that the VAFS does little in terms of helping us understand the severity of fatigue in PD, we hope that the results from this pilot study will stimulate further investigations that lead to a better understanding of the mystifying sensation of fatigue in Parkinson’s disease and its impact on quality of life.
